# Students, Research, and the Health of Appalachia

**DOI:** 10.13023/jah.0301.01

**Published:** 2021-01-24

**Authors:** Douglas Scutchfield

**Affiliations:** University of Kentucky

**Keywords:** Appalachia, research, students, mentors, publication

## Abstract

The notion of publication in the peer-reviewed literature out of your doctoral or master’s thesis/dissertation or capstone is a characteristic of those who choose a career in the academy. This paper illustrates my pleasure by reflecting a student taking the additional step in research achievement by publishing results that contribute new knowledge to evidence-driven research and practice.

In this issue of the *Journal of Appalachian Health* is an article by Dannell Boatman, entitled *Appalachian Caregiver Perspectives on Childhood Gun Safety in the Home*. This paper highlights the work she did for her doctoral dissertation. Like many in the public health workforce, Dr. Boatman pursued and received the doctoral degree via online education. I applaud that achievement and recognize that after the doctorate, Dannell has started working on a master’s in public health, pursuing another graduate degree that will further enhance her contribution to her work in cancer control. She also represents other public health workers who come to the discipline in a variety of ways and who remain employed while pursuing their graduate degrees.

The recognition of Dr. Boatman’s article is also the first time the Journal has formally acknowledged such work. We may have published others, but we are now formally recognizing these student contributions to actionable research resulting in improving the health status of the Appalachian population. The formal recognition also includes the acknowledgment of research mentors who have supported Dr. Boatman’s research and its publication. Having personally worked closely mentoring young researchers, I know it is both challenging and uplifting as we help those who would follow us to understand the importance of contributions of new knowledge to our healing efforts.

The notion of publication in the peer-reviewed literature out of your doctoral or master’s thesis/dissertation or capstone is a characteristic of those who choose a career in the academy. This paper illustrates my pleasure by reflecting a student taking the additional step in research achievement by publishing results that contribute new knowledge to evidence-driven research and practice.

The editorial team hopes that new graduate degree recipients and their mentors will consider the Journal as a potential home for the publication of these fresh research efforts, efforts that provide evidence to assist our pursuit of the most effective means to contribute to a healthy and thriving Appalachia.

